# Pediatric Lung Transplantation Following Non-Transplant Cardiac Surgery: A Contemporary Analysis

**DOI:** 10.1007/s00408-026-00893-z

**Published:** 2026-05-06

**Authors:** Wonshill Koh, JangDong Seo, Todd Jenkins, Don Hayes

**Affiliations:** 1 Heart Institute, Cincinnati Children’s Hospital Medical Center, Cincinnati, 3333 Burnet Avenue, Cincinnati, OH USA; 2 Department of Pediatrics, University of Cincinnati College of Medicine, Cincinnati, OH USA; 3 Division of Biostatistics and Epidemiology, Cincinnati Children’s Hospital Medical Center, Cincinnati, OH USA; 4 Division of Pulmonary Medicine, Cincinnati Children’s Hospital Medical Center, Cincinnati, OH USA

## Abstract

**Purpose:**

More children are undergoing congenital or non-congenital cardiac surgery today which can impact outcomes for subsequent thoracic surgery. However, post-lung transplant (LTx) outcomes of children with previous cardiac surgery are unknown, so we explored this important issue using a publicly available database.

**Methods:**

A retrospective analysis was performed using the Scientific Registry of Transplant Recipients (SRTR). First-time pediatric LTx candidates without and with history of prior cardiac surgery, excluding previous cardiothoracic transplantation, from 2003 to 2024 were enrolled into our study. Univariate analyses, multivariable Cox regression, and Kaplan–Meier plots were performed for a comprehensive analysis.

**Results:**

We identified 1333 and 144 LTx candidates without and with prior cardiac surgery (52 with congenital surgery, 92 with non-congenital surgery) with more children with cardiac surgery being listed for LTx over time. There were 811 LTx recipients without prior cardiac surgery compared to 63 with prior cardiac surgery (14 congenital, 49 non-congenital). Children with prior congenital cardiac surgery were much younger, and pulmonary vascular disease (PVD) was the most common indication for LTx. Prior non-congenital cardiac surgery did not negatively impact short- or long-term post-LTx outcomes in children. However, history of congenital cardiac surgery was associated with high waitlist mortality (31% compared to 15% (no surgery) and 21% (non-congenital surgery), p < 0.001) and worse long-term outcomes (HR 1.89; 95% CI 1.01, 3.53, p = 0.048).

**Conclusions:**

There is an increasing number of children with previous cardiac surgery undergoing LTx especially in the setting of congenital heart disease with subsequent PVD.

## Key Message

Key words: pediatric, congenital heart disease, lung transplant, cardiac surgery

## Introduction

There has been sustained improvement in outcomes for children after lung transplant (LTx) [1–7]. As outcomes improve for these children, a marked shift regarding the historical indications for LTx has occurred, where cystic fibrosis (CF) is no longer the leading indication with other pulmonary conditions including restrictive lung diseases and pulmonary vascular disease (PVD) becoming more common reasons for consideration [1, 3–5, 8]. Similarly, combined heart–lung transplantation (HLTx) has evolved as it remains to be an important surgical treatment option for a select group of adult and pediatric patients with cardiopulmonary failure with outcomes following similar improvements for LTx alone [9–11]. In adult patients with Eisenmenger syndrome who may undergo LTx with or without cardiac surgery or combined HLTx, LTx was associated with superior outcomes compared to HLTx for patients with atrial septal defects and equivalent outcomes for those with ventricular septal defects [12]. Even though the number of cardiac surgeries for congenital and non-congenital indications has increased globally in children, there is scarce literature investigating LTx outcomes for children who have previously underwent cardiac surgery for either congenital or non-congenital reasons [13–16]. Due to the paucity of pediatric literature, we performed a multi-institutional registry analysis to investigate the prevalence of LTx among children with a history of congenital or non-congenital cardiac surgery and examined waitlist and LTx outcomes.

## Methods

### Data Collection

A retrospective analysis was performed using the Scientific Registry of Transplant Recipients (SRTR). The study was approved by the Cincinnati Children’s Hospital Medical Center Institutional Review Board. The registry database was queried from 2003 to 2024 for first-time pediatric LTx candidates under the age of 18 with known history of prior cardiac surgery excluding previous cardiothoracic transplant. Patients with unknown status of prior cardiac surgery, missing entry for underlying diagnosis/reason for waitlist removal/transplant or listing date, or multi-listing with other solid organs were also excluded. Extracted data variables included demographic characteristics, indication for transplant listing, body mass index (BMI), extracorporeal mechanical oxygenation (ECMO) and mechanical ventilation (MV) support status at the time of waitlist and transplant, creatinine level, length of hospital stay, and post-transplant survival status. Diagnosis was grouped as PVD (primary/secondary pulmonary hypertension and pulmonary veno-occlusive disease), obstructive disease including CF and bronchiolitis obliterans, restrictive lung disease (interstitial lung disease and acute respiratory distress syndrome), and others/congenital heart disease (CHD) including unspecified, myopathy, other congenital malformation, etc. Listing and transplant era were stratified for two 10-year period eras: 2003—2013 and 2014—2024.

### Statistical Analysis

Descriptive statistics for continuous variables were described using medians with interquartile ranges (IQR) and frequencies with percentages for categorical variables. Chi-square tests, Fisher’s exact tests, and Wilcoxon rank-sum tests were used to test differences in demographic and clinical characteristics according to history of previous thoracic surgery. Cox multivariable regression was used to identify risk factors for 1-year and 10-year post-LTx outcomes by computing adjusted hazard ratios (HR) and 95% confidence interval (CI) for death or re-transplant. The proportional hazards assumption was checked by plots of the Schoenfeld residuals. Covariates for the Cox proportional hazard regression included history of prior cardiac history, age at listing, race, diagnosis, ECMO/MV status, and transplant era. Kaplan–Meier curve was generated to compare the outcomes on transplant free survival after LTx between those with prior congenital or non-congenital cardiac surgery and those without. Follow-up time was calculated from the date of the LTx until the date of death, re-transplant, or censoring. Patients alive and not undergoing a re-transplant were censored at the last date of follow-up. Two-sided p values < 0.05 were considered statistically significant. All statistical analyses were performed using R version 4.5.2 statistical software. Cox regression models were performed using the coxph function in the R package survival (v3.8.6). Kaplan–Meier curves were generated using the survival (v3.8.6) and survminer (v0.5.2) packages.

## Results

### Clinical Characteristics for LTx Candidates with and Without Prior cardiac Surgery

There were 1477 LTx candidates eligible for our analysis with 1333 without and 144 with prior cardiac surgery (Table 1). 52 out of 144 (36%) had congenital cardiac surgery, and 92 (64%) had non-congenital cardiac surgery. The median age for LTx candidates was lowest for those with congenital surgery (4 vs 8.5 non-congenital surgery vs 13 without surgery, p < 0.001) with 55% being under the age of 6. Obstructive lung disease was the leading cause of LTx for those without cardiac surgery (60%) while PVD was the leading indication for children with congenital surgery (79%) (p < 0.001). More LTx candidates with congenital cardiac surgery required MV at the time of waitlist (29% vs 18% non-congenital surgery vs 15% without surgery, p = 0.03) while more children with non-congenital cardiac surgery required ECMO support at the time of waitlist (15% vs 5.8% congenital surgery vs 6% without surgery, p = 0.006). More than half of LTx candidates without cardiac surgery (61%) or with non-congenital surgery (53%) were successfully bridged to LTx while only 27% of children with congenital cardiac surgery received LTx with the highest rate of waitlist mortality (31%, p < 0.001). There was an increase in the number of LTx listing for children with congenital cardiac surgery in the recent era of 2014—2024 (54%) compared to the previous era of 2003—2013 (46%). Between the two eras, the number of LTx listings was similar for children with non-congenital surgery (51% vs 49%) while LTx listings for children without cardiac surgery much decreased (65% vs 35%) (p = 0.001).

**Table 1:** Clinical characteristics for pediatric LTx candidates on waitlist without and with prior cardiac surgery

Variables	Total N = 1477 History of cardiac surgery			p-value
	**No** N = 1333	**Yes** N = 144		
		**Congenital** N = 52	**Non-congenital** N = 92	
Gender				**0.002**
F	797 (60%)	20 (38%)	46 (50%)	
M	536 (40%)	32 (62%)	46 (50%)	
Age (year)	13 (8, 16)	4 (0, 13)	8.5 (1, 14)	** < 0.001**
Age group (year)				** < 0.001**
< 6	252 (19%)	29 (55%)	38 (41%)	
6—11	290 (22%)	6 (11%)	19 (21%)	
12—17	791 (59%)	17 (33%)	35 (38%)	
Race				**0.004**
White	1154 (87%)	41 (79%)	72 (78%)	
African American	117 (8.8%)	4 (7.7%)	9 (9.8%)	
Asian	33 (2.5%)	5 (9.6%)	9 (9.8%)	
Other/CHD	29 (2.2%)	2 (3.8%)	2 (2.2%)	
Diagnosis				** < 0.001**
PVD	291 (22%)	41 (79%)	31 (34%)	
Obstructive	794 (60%)	1 (2%)	34 (37%)	
Restrictive	153 (11%)	0	19 (21%)	
Other	95 (7%)	10 (19%)	8 (8%)	
BMI (kg/m^2^)	17 (15, 19)^a^	16 (15, 18)	16 (14, 18)	**0.005**
Creatinine (mg/dL)	0.4 (0.3, 0.6)^b^	0.3 (0.2, 0.6)^c^	0.3 (0.2, 0.48)^d^	** < 0.001**
MV at the time of waitlist	206 (15%)	15 (29%)	17 (18%)	**0.03**
ECMO at the time of waitlist	80 (6.0%)	3 (5.8%)	14 (15%)	**0.006**
Reason for waitlist removal				** < 0.001**
Transplant	811 (61%)	14 (27%)	49 (53%)	
Died on waitlist	202 (15%)	16 (31%)	19 (21%)	
Medically unsuitable	61 (4.6%)	1 (1.9%)	8 (8.7%)	
Improved	92 (6.9%)	4 (7.7%)	6 (6.5%)	
Died during transplant	4 (0.3%)	0 (0%)	0 (0%)	
Other	163 (12%)	17 (33%)	10 (11%)	
Listing Era				**0.001**
2003—2013	860 (65%)	24 (46%)	47 (51%)	
2014—2024	473 (35%)	28 (54%)	45 (49%)	

Categorical variables as *n* (%) and Continuous variable as median (IQR). ^a^n = 1332, ^b^n = 888, ^c^n = 29, ^d^n = 51

### Clinical Characteristics of LTx Recipients with and Without Cardiac Surgery

Of the children who received LTx, 811 had not undergone prior cardiac surgery and 63 had (14 with congenital and 49 with non-congenital) (Table 2). Among 14 children with prior congenital cardiac surgery, 3 were palliated while 11 had complete corrective cardiac surgery. The median age was again lowest for the children with congenital cardiac surgery (age 6) with 50% of them being under the age of 6 while 63% and 45% of LTx recipients were adolescents for those without cardiac surgery and with non-congenital surgery, respectively (p = 0.001). Obstructive lung disease was again the leading LTx indication for children without cardiac surgery (66%) while PVD was the most common indication for LTx for those with congenital cardiac surgery (79%) (p < 0.001). There was similar distribution among diagnoses (33% obstructive, 31% restrictive, and 29% PVD) for recipients with non-congenital cardiac surgery. More children with non-congenital surgery (28%) required MV at the time of LTx with 64% of them needing MV after being waitlisted (p = 0.001). Also, more children with non-congenital cardiac surgery were bridged to LTx on ECMO (18% vs 14% for congenital surgery vs 7.8% for without surgery, p = 0.03). The overall number of pediatric LTxs for those without cardiac surgery decreased (42% 2014—2024 vs 58% 2003—2013). The LTx numbers for those with congenital cardiac surgery remained the same (50% and 50%) while the LTx numbers for children with non-congenital cardiac surgery increased (55% 2014—2024 vs 45% in 2003—2013).

**Table 2:** Clinical characteristics for pediatric LTx recipients without and with prior cardiac surgery

Variables	Total N = 874 History of cardiac surgery			p-value
	**No** N = 811	**Yes** N = 63		
		**Congenital** N = 14 (palliative surgery n = 3/corrective surgery n = 11)	**Non-congenital** N = 49	
Gender				0.39
F	482 (59%)	6 (43%)	27 (55%)	
M	329 (41%)	8 (57%)	22 (45%)	
Age (year)	14 (8, 16)	6 (1, 13.7)	10 (1, 16)	**0.006**
Age group (year)				**0.001**
< 6	139 (17%)	7 (50%)	17 (35%)	
6—11	163 (20%)	1 (7%)	10 (20%)	
12—17	509 (63%)	6 (43%)	22 (45%)	
Diagnosis				** < 0.001**
PVD	128 (16%)	11 (79%)	14 (29%)	
Obstructive	539 (66%)	1 (7%)	16 (33%)	
Restrictive	92 (11%)	0 (0%)	15 (31%)	
Other/CHD	52 (6.4%)	2 (14%)	4 (8%)	
Race				**0.006**
White	714 (88%)	11 (79%)	38 (78%)	
African American	65 (8.0%)	2 (1.4%)	5 (10%)	
Asian	14 (1.7%)	1 (19.6%)	5 (10%)	
Other	18 (2.2%)	0 (0%)	1 (2%)	
Hospital Stay (days)	20 (14, 34)^a^	32 (14, 46)	25 (15, 55)	0.07
Creatinine (mg/dL)	0.4 (0.3, 0.59)^b^	0.45 (0.29, 0.57)	0.38 (0.21, 0.51)	0.30
MV at the time of LTx	151 (19%)	2 (14%)	14 (28%)	0.20
from the time of waitlist	105 (70%)	2 (100%)	5 (36%)	0.84
after the waitlist	46 (30%)	0 (0%)	9 (64%)	**0.001**
ECMO at the time of LTx	63 (7.8%)	2 (14%)	9 (18%)	**0.03**
from the time of waitlist	42 (67%)	1 (50%)	5 (56%)	0.31
after the waitlist	21 (33%)	1 (50%)	4 (44%)	0.05
Transplant era				0.17
2003—2013	470 (58%)	7 (50%)	22 (45%)	
2014—2024	341 (42%)	7 (50%)	27 (55%)	

Categorical variables as *n* (%) and Continuous variable as median (IQR). ^a^n = 807, ^b^n = 807

### Multivariable Analysis and Kaplan–Meier Survival Analysis for post-LTx Outcomes

Cox multivariable regression analysis showed that combined congenital and non-congenital cardiac surgery was not a risk factor for worse post-LTx outcome in both short-term (HR 1.42; 95% CI 0.79, 2.54; p = 0.24) and long-term (HR 1.22; 95% CI 0.87, 1.71; p = 0.26) (Table 3 and 4). When looking at surgery types separately, neither congenital (HR 1.36; 95% CI 0.41, 4.47; p = 0.61) nor non-congenital (HR 1.43; 95% CI 0.75, 2.73; p = 0.28) appeared to have any statistically significant impact on 1-year post-LTx outcome (Table 3). The only risk factor associated with worse 1-year post-LTx outcome was MV at the time of LTx (HR 2.12; 95% CI 1.38, 3.25; p = 0.001) but it did not have any negative impact on 10-year post LTx outcome (HR 0.92; 95% CI 0.71, 1.19; p = 0.53). For 10-year post-LTx outcomes, congenital cardiac surgery appeared to be a risk factor for worse outcome (HR 1.89; 95% CI 1.01, 3.53; p = 0.048) while non-congenital cardiac surgery had no effect (HR 1.07; 95% CI 0.72, 1.58; p = 0.75) (Table 4). Two other risk factors that were associated with worse 10-year post-LTx outcome were adolescence age (HR = 1.26; 95% CI:1.03, 1.53; p < 0.02) and African American race (HR = 1.63; 95% CI:1.18, 2.23; p = 0.003). The recent era 2014—2024 was associated with improved 10-year post-LTx outcome (HR = 0.79; 95% CI:0.65, 0.96; p = 0.02).

**Table 3:** Hazard Ratios (HR) and 95% Confidence Intervals (CI) for 1-year post-LTx survival according to risk factors from the cox regression model, N = 873

Characteristic	HR	95% CI	p-value
*Prior cardiac surgery*			
*No (ref)*			
Yes	1.42	0.79, 2.54	0.24
Yes (Congenital)	1.36	0.41, 4.47	0.61
Yes (Non-congenital)	1.43	0.75, 2.73	0.28
*Age group*			
** < 12 (ref)**			
12—17	0.91	0.61, 1.35	0.63
*Race*			
**White (ref)**			
African American	1.17	0.64, 2.13	0.61
Asian	1.93	0.82, 4.54	0.13
Other	0.54	0.13, 2.25	0.4
*Diagnosis*			
**PVD (ref)**			
Obstructive	0.7	0.44, 1.12	0.14
Restrictive	0.6	0.31, 1.15	0.12
Other/CHD	0.58	0.27, 1.25	0.16
*MV at the time of LTx*			
**No (ref)**			
Yes	2.12	1.38, 3.25	** < 0.001**
*ECMO at the time of LTx*			
**No (ref)**			
Yes	1.42	0.77, 2.62	0.26

**Table 4:** Hazard Ratios (HR) and 95% Confidence Intervals (CI) for 10-year post-LTx survival according to risk factors from the cox regression model, N = 873

Characteristic	HR	95% CI	p-value
*Prior cardiac surgery*			
**No (ref)**			
Yes	1.22	0.87, 1.71	0.26
Yes (congenital)	1.89	1.01, 3.53	**0.048**
Yes (non-congenital)	1.07	0.72, 1.58	0.75
*Age group*			
* < 12 (ref)*			
12—17	1.26	1.03, 1.54	**0.02**
*Race*			
*White (ref)*			
African American	1.63	1.18, 2.23	**0.003**
Asian	1.14	0.60, 2.16	0.70
Other	1.17	0.60, 2.27	0.65
*Diagnosis*			
**PVD (ref)**			
Obstructive	1.05	0.80, 1.37	0.73
Restrictive	1.04	0.73, 1.47	0.82
Other/CHD	0.84	0.54, 1.32	0.46
*MV at the time of LTx*			
**No (ref)**			
Yes	0.92	0.71, 1.19	0.53
*ECMO at the time of LTx*			
**No (ref)**			
Yes	1.25	0.86, 1.80	0.24
*Transplant era*			
**2003—2013 (ref)**			
2014—2024	0.79	0.65, 0.96	**0.02**

**Fig. 1:**
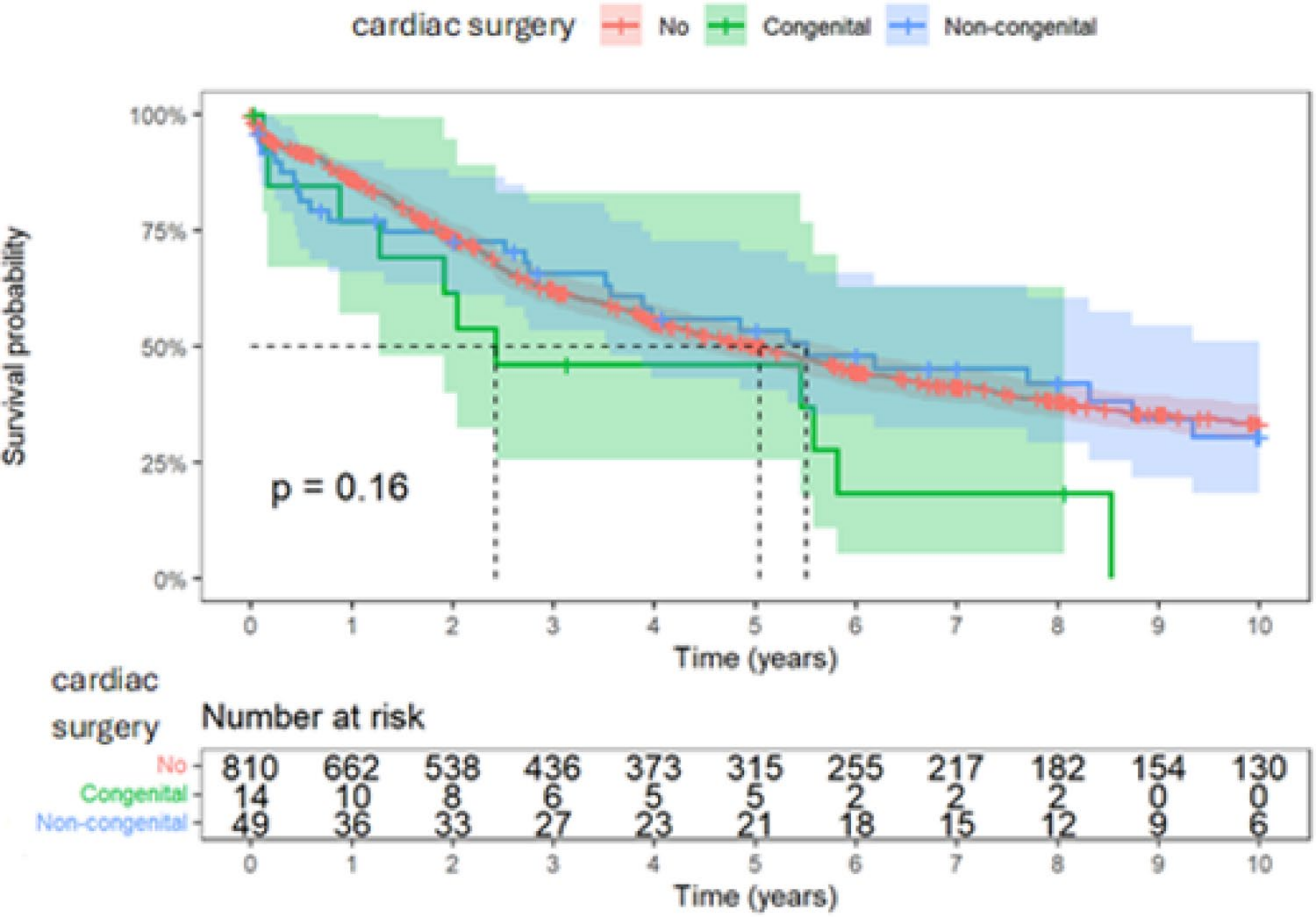
Kaplan–Meier estimates of survival probability for pediatric LTx recipients with no prior cardiac surgery, congenital cardiac surgery, and non-congenital surgery. Shaded areas are 95% confidence intervals

There was similar trend seen with Kaplan–Meier analysis (Fig. 1). Although transplant-free 10-year post-LTx survival outcomes among three groups did not achieve a statistical significance, we saw that no children with congenital heart surgery achieved transplant-free survival beyond 8 years (transplant-free survival 86% vs 77% vs 77% at 1 year, 62% vs 46% vs 65% survival at 3 year, 33% vs 0% vs 30% at 10 year for no surgery vs congenital cardiac surgery vs non-congenital cardiac surgery).

## Discussion

The key findings for the current study include that listing for LTx among children with a history of cardiac surgery, especially with congenital cardiac surgery, has increased and previous congenital or non-congenital cardiac surgery did not appear to have significant impact on short-term post-LTx outcomes. However, our results suggest that congenital cardiac surgery is associated with increased waitlist mortality and worse long-term outcomes.

Globally, the number of children being listed for and undergoing LTx has decreased, largely in the CF patient population due to the development of innovative CF modulator therapies [1, 3, 5, 7]. In comparison, the current study identified a gradual increase in the listing of children with prior congenital cardiac surgery for LTx in the United States (from 46% in 2003—2013 to 54% in 2014—2024). Conversely, the need for LTx in children with previous non-congenital cardiac surgery has remained consistent across these two eras (~ 50%). While more children with congenital cardiac surgery have required LTx, they experienced the highest mortality on the waitlist, 31% with only 27% successfully receiving LTx. A key factor contributing to the high waitlist mortality in this group is the age distribution of this subgroup (55% of children under the age of 6). Historically, younger children have a higher mortality rate on the waitlist for LTx, directly related to donor shortage and the challenges in donor-recipient lung size matching [1, 2, 17]. Notably, the majority (> 50%) of children without cardiac surgery or with non-congenital cardiac surgery received LTx with most of them being adolescents.

The most common indication for LTx among children with prior congenital cardiac surgery was PVD (79% for candidates and recipients). In our opinion, this finding supports the close association between CHD and PVD in these children and suggests that PVD in this population is progressive [18–20]. Alas, the registry data used for our analysis prohibits the discernment of key underlying clinical details including identification of cardiac lesions or previous surgical procedures performed along with the clinical course that may result in PVD for these children. However, it appears that progressive PVD in children with prior congenital cardiac surgery may be an important factor in the rising numbers of LTx being performed for PVD in the pediatric population [1–5, 8]. Conversely, our findings that more children are undergoing LTx for PVD in the setting of congenital heart disease in the modern era is likely the reason why fewer children are undergoing HLTx as reported by others [9, 10]. Advances in the surgical and medical management of LTx contributed to the reduced need for HLTx in patients with PVD [5, 9, 21]. It also appears that congenital cardiac surgery is reducing the need for as well, but the need for LTx remains due to the associated PVD. We believe this needs further exploration with multi-institutional studies that provide more granular data would help tease out this very important question with registry data not permitting a more in-depth analysis.

In the modern era, listing numbers for LTx increased for children with congenital cardiac surgery. The number of actual LTx among these children remained the same across different eras which is likely influenced by the higher waitlist mortality for this group. Meanwhile, the number of LTx among children with non-congenital cardiac surgery has increased and we identified a greater need for MV or ECMO support at the time of LTx in children with non-congenital heart surgery. Due to the design of the current study and the limited dataset, we cannot identify the reasoning for the differences in MV and ECMO support, but we postulate that children requiring non-congenital cardiac surgeries are more likely to present with acute cardiac or respiratory compromise secondary to infection or trauma or possibly chronic complications from previous treatment of malignancy.

Importantly, the current study found that prior congenital and non-congenital cardiac surgery did not negatively impact short- or long-term post-LTx outcomes in children. However, when we investigated these separately, we identified that prior congenital cardiac surgery was associated with worse long-term outcomes. There was not a significant difference in overall survival based on our Kaplan–Meier survival analysis between children without prior surgery and those with congenital or non-congenital cardiac surgery, but this analysis was limited by our small cohort with no children in the congenital cardiac surgery cohort achieving transplant-free survival past 8 years following LTx. The most common cause of mortality was graft failure for children with both congenital and non-congenital cardiac surgery. Further study to validate the impact of outcomes will require a larger cohort size and additional detailed clinical information.

Our multivariable analysis elicited key findings. We identified that MV at the time of LTx was associated with increased short-term mortality risk at 1-year post-LTx but not long-term at the 10- year timepoint. These findings are consistent with previous studies that also show pre-transplant MV is associated with short-term post-LTx mortality in both children and adults [22–24]. The need for EMCO support at time of LTx had no impact on post-LTx outcome in our analysis, but adolescent age and African American race continued to be risk factors for increased long-term post-LTx mortality as previously reported [1, 3, 7, 25–27]. LTxs performed in the more recent era were associated with improved long-term post-LTx outcomes, which supports that the management of these patients is improving over time [1, 3, 7, 21].

The current study has several limitations arising from retrospective design using large multi-institutional registry data. Limited by variables available in the registry, data was incomplete for several variables. The registry does not provide exact diagnosis of original congenital or non-congenital lesions or types/dates of cardiac surgery other than noting history of congenital or non-congenital cardiac surgery. Other variables not available are modality/duration of ECMO or duration of MV. Furthermore, a relatively smaller pediatric sample size, especially for those with congenital cardiac surgery, limits the statistical power of our study. However, the current study draws results from a large, multi-institutional registry database providing novel insights into this subset of these high-risk children.

## Conclusion

There is an increased number of children with previous cardiac surgery needing LTx especially those with congenital lesions and PVD. Although prior cardiac surgery appears to have acceptable post-LTx outcomes in general, children with prior congenital cardiac surgery experience higher waitlist mortality and worse long-term outcomes. Therefore, this disparity experienced by this subset of pediatric candidates should be addressed to ensure that donor allocation policies are in place to allow equitable access to donor organs for them. Finally, longitudinal, multi-institutional studies are needed to better understand best approaches for timing of referral for LTx evaluation, determination of transplant candidacy, and post-transplant management.

## Data Availability

The data used for this study has been supplied by the Scientific Registry of Transplant Recipients (SRTR). The content is the responsibility of the authors alone and does not necessarily reflect the views or policies of the SRTR or the US Government.
